# Standardization of ^123^I-*meta*-iodobenzylguanidine myocardial sympathetic activity imaging: phantom calibration and clinical applications

**DOI:** 10.1007/s40336-017-0230-2

**Published:** 2017-05-04

**Authors:** Kenichi Nakajima, Derk O. Verschure, Koichi Okuda, Hein J. Verberne

**Affiliations:** 1 Department of Nuclear Medicine, Kanazawa University, 13-1 Takara-machi, Kanazawa, 920-8641 Japan; 2 Department of Nuclear Medicine, Academic Medical Center, University of Amsterdam, Amsterdam, The Netherlands; 3 Department of Cardiology, Zaans Medical Center, Zaandam, The Netherlands; 4 Department of Physics, Kanazawa Medical University, Uchinada, Kahoku, Japan

**Keywords:** Quantification, Heart-to-mediastinum ratio, Calibration phantom, Conversion coefficient, Collimator

## Abstract

**Purpose:**

Myocardial sympathetic imaging with ^123^I-*meta*-iodobenzylguanidine (^123^I-*m*IBG) has gained clinical momentum. Although the need for standardization of ^123^I-*m*IBG myocardial uptake has been recognized, the availability of practical clinical standardization approaches is limited. The need for standardization includes the heart-to-mediastinum ratio (HMR) and washout rate with planar imaging, and myocardial defect scoring with single-photon emission computed tomography (SPECT).

**Methods:**

The planar HMR shows considerable variation due to differences in collimator design. These camera–collimator differences can be overcome by cross-calibration phantom experiments. The principles of these cross-calibration phantom experiments are summarized in this article. ^123^I-*m*IBG SPECT databases were compiled by Japanese Society of Nuclear Medicine working group. Literature was searched based on the words “^123^I-*m*IBG quantification method”, “standardization”, “heart-to-mediastinum ratio”, and its application to “risk model”.

**Results:**

Calibration phantom experiments have been successfully performed in Japan and Europe. The benefit of these cross-calibration phantom experiments is that variation in the HMR between institutions is minimized including low-energy, low–medium-energy and medium-energy collimators. The use of myocardial ^123^I-*m*IBG SPECT can be standardized using ^123^I-*m*IBG normal databases as a basis for quantitative evaluation. This standardization method can be applied in cardiac event prediction models.

**Conclusion:**

Standardization of myocardial ^123^I-*m*IBG outcome parameters may facilitate a universal implementation of myocardial ^123^I-*m*IBG scintigraphy.

## Introduction

Heart failure (HF) is a life-threatening disease affecting approximately 26 million people worldwide [1]. The cardiac sympathetic system is an important neurohormonal compensation mechanism in the pathogenesis of chronic heart failure (CHF). Patients with CHF have increased cardiac sympathetic activity with increased exocytosis of norepinephrine (NE) from the presynaptic vesicles and impaired NE re-uptake via the norepinephrine transporter in the sympathetic terminal nerve axons. This results in increased NE levels in the synaptic cleft. Initially, β-adrenergic receptor stimulation by increased synaptic NE levels helps to compensate for impaired myocardial function. However, long-term NE excess has detrimental effects on myocardial structure and gives rise to a downregulation and availability of post-synaptic β-adrenergic receptor. This leads to left ventricular remodeling and is associated with increased mortality and morbidity in CHF. CHF presents high mortality rates and around 50% of deaths are related to sudden death. The European data (ESC-HF pilot study) demonstrate that 12-month all-cause mortality rates for hospitalized and stable/ambulatory CHF patients were 17 and 7%, respectively [2]. The majority of these deaths are caused by progression of HF, lethal arrhythmia and sudden cardiac death.

Myocardial ^123^I-*meta*-iodobenzylguanidine (^123^I-*m*IBG) scintigraphy has the unique characteristic of reflecting myocardial sympathetic activity and has been used in treating many kinds of cardiac diseases [3–6]. In particular, in patients with CHF, myocardial ^123^I-*m*IBG scintigraphy is the most common indication in Japan, Europe and the United States [7, 8]. However, myocardial ^123^I-*m*IBG is also widely used for the diagnosis of Lewy body-related disorders [9, 10], and recently the number of ^123^I-*m*IBG studies for neurological indications has exceeded cardiac applications in Japan [11]. While various quantitation methods for measuring myocardial uptake have been used, the simplest and most practically used index has been heart-to-mediastinum ratio (HMR) [12]. However, there are inter-institutional variations in HMR, even for normal values. Especially the choice of collimator introduces considerable variations [13, 14]. Therefore, the need for standardization of ^123^I-*m*IBG myocardial uptake has been recognized. However, the availability of practical clinical standardization approaches is limited. To overcome this variation and to unify HMRs from various data acquisition systems, a cross-calibration phantom was developed [15, 16]. In this article, the general idea behind this cross-calibration method is reviewed. Literature was searched using the following words: “quantification”, “^123^I-*m*IBG”, “heart-to-mediastinum ratio”, “washout rate”, “standardization”, “collimators”, and “phantoms”. In addition, the use of this standardization method in cardiac event prediction models is included. Last but not least, standardization methods for myocardial ^123^I-*m*IBG single-photon emission computed tomography (SPECT) are discussed.

## What should be standardized?

Since the introduction of the HMR in patients with CHF, it has been widely used as a practical method to reflect myocardial sympathetic activity. The need for standardization in ^123^I-*m*IBG studies has been recognized for quantitation methods [17–20]. The HMR is a practical but crude parameter and is calculated by an average count ratio between heart and mediastinum. However, the practical simplicity of the HMR does not necessarily result in its uniformity, and various factors are known to cause variations in the HMR [7, 12]. Since the HMR is determined by an averaged count ratio of heart and mediastinum, the location, size, and shape of the region of interest (ROI) could be a factor of the variations. Although in clinical practice such variations between hospitals exist, the reproducibility of the HMR is generally believed to be good [21, 22]. However, some variations still exist depending on ROI setting methods even around the prognostic threshold of HMR (1.6), and predefined or semiautomatic methods for defining ROI are preferable [22–24]. Another critical factor is related to camera–collimator combinations, in particular, regarding low-energy (LE) and medium-energy (ME) collimators [13–15]. The HMR derived from ME collimators is higher than that from LE collimators. The Japanese Society of Nuclear Medicine working group (JSNM-WG) normal databases showed an average HMR of 2.5 (range of mean ± 2 standard deviation: 1.9–3.1) and 3.0 (range 2.0–4.3) for the LE and ME collimators, respectively [25]. Although the use of ME collimators is advocated [12], many hospitals still use LE collimators because of limited availability of ME collimators. Moreover, especially in Japan, as the use of ^123^I-labeled tracers for brain perfusion and myocardial imaging is common, various types of collimators have specifically been designed for these ^123^I-labeled radiopharmaceuticals. One may argue that calculated HMRs are consistent if all institutions were to use the same type of collimators. However, collimator specifications such as hole diameter, length and septal thickness may significantly differ between manufacturers despite the fact that these collimators have similar names. The impact of these variations in collimators on the HMR is best illustrated by a Japanese multicenter phantom study [16]. In this study variations in the phantom identified an HMR of 1.6 for the same LE high-resolution (LEHR) collimators which ranged in measured values between 1.50 and 1.74 [26].

## Structure of the phantom and experiments

**Fig. 1:**
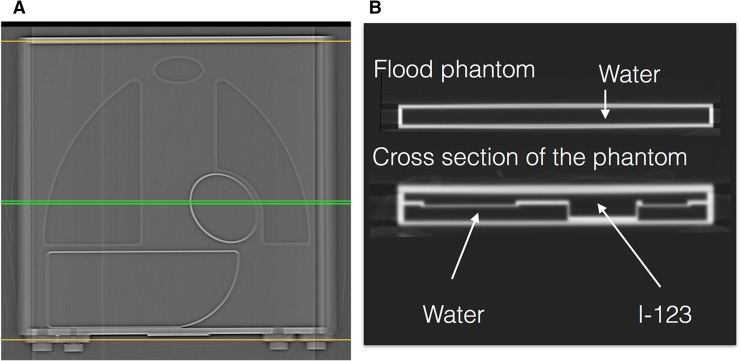
Cross-calibration phantom with a scout *anterior view* (**a**) and transverse X-ray CT cross section (**b**) illustrating the structure of the phantom and the different simulated organs

To cross-calibrate camera–collimator systems in various hospitals, a calibration phantom was introduced and successfully applied in 225 conditions in Japan [16], and subsequently validated in Europe in 210 conditions [27]. Additionally, as of 2016 nearly 1300 calibration phantom experiments for additional collimators were performed using the same acquisition protocol. The calibration phantom was designed for planar acquisitions for the calculation of the HMR only (Fig. 1). Although it cannot be used for SPECT acquisitions, the design allows for acquiring images from anterior and posterior views resulting in a different HMR. The phantom is made of acryl with two separate compartments: one compartment can be filled with a ^123^I solution (111–185 MBq) and the second compartment can be filled with water. Mediastinum, liver, lung and heart are simulated by variation in acryl thickness that separates the two compartments. As ^123^I can be distributed homogeneously in one compartment, adjustment for ^123^I concentrations is not necessary. Both sides of the phantom (i.e., anterior and posterior) can be imaged resulting in a different HMR. Typically the protocol used for the described phantom experiments included a 256 × 256 matrix and an acquisition duration of 5 min [16, 27]. The energy window was centered at 159 keV with a range of 15–20%.

## Conversion coefficient for collimators

**Fig. 2:**
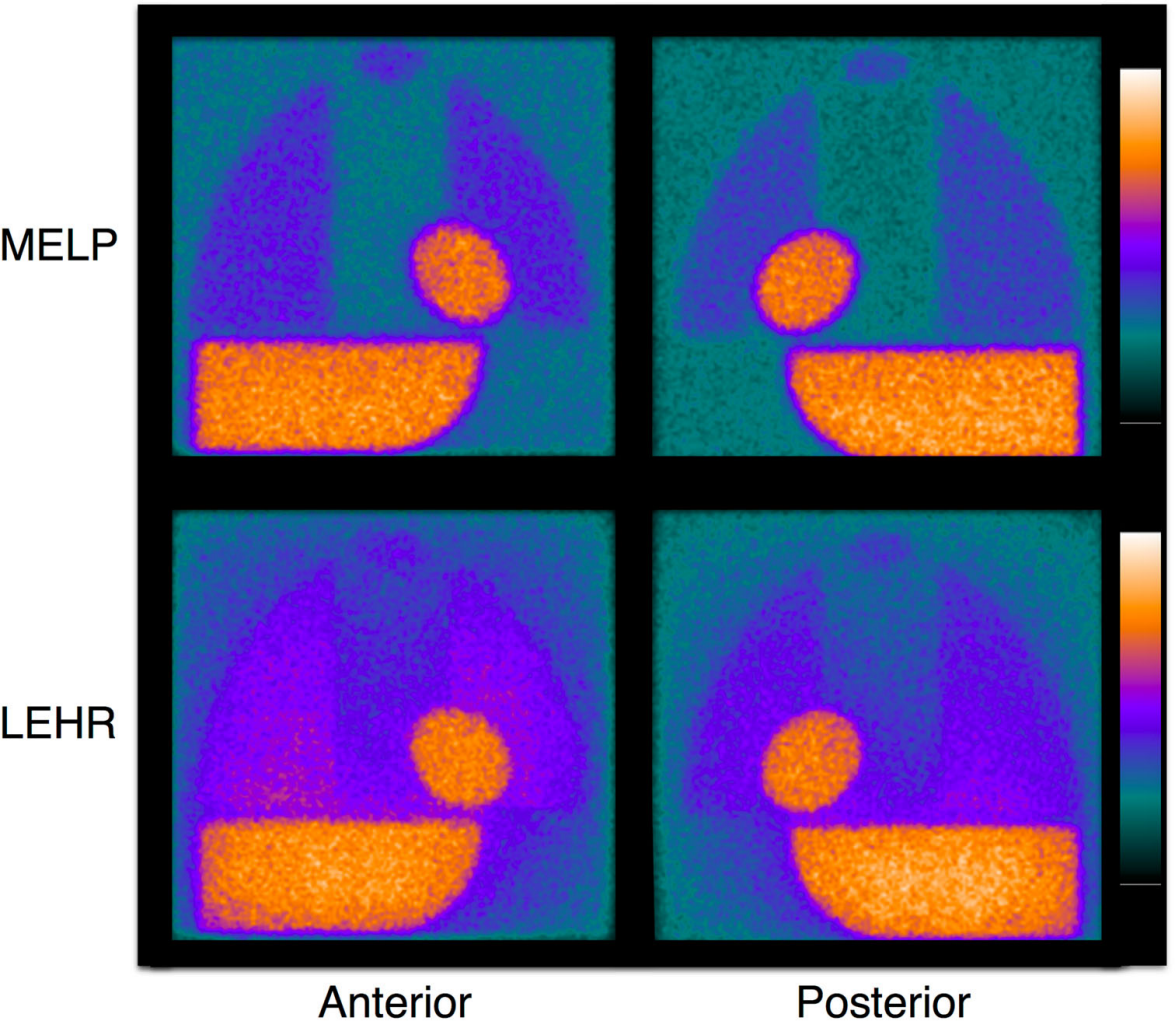
Anterior and posterior phantom images obtained with medium-energy low-penetration (MELP) and low-energy high-resolution (LEHR) collimators. Difference in background count due to septal penetration and scatter can be clearly seen

In the initial experiments, four phantom types were used, and eight HMRs could be calculated to make a linear regression line between two camera systems [15]. The reference HMR was calculated mathematically using attenuation factors ranging from 1.55 to 4.48 (Fig. 2). Thereafter, the number of phantoms was first reduced to two and finally to only one. The reduction of phantoms was based on the fact that the variation in regression lines between different camera systems did not change with the lower number of phantoms, ultimately leading to only one phantom (i.e., with 2 different HMRs) [15]. The slope K was defined as the conversion coefficient (CC) that allows for the conversion of measured values to reference values. This mathematically calculated HMR was used as the reference or gold standard, for which attenuation in the acrylic plates and water was calculated. Although HMRs from any two camera–collimator systems can be cross-calibrated, we extended the idea to calculate one single standardized condition, so that any institution can calculate HMRs using essentially the same scale. Since the European proposal advocated the use of ME collimators [12], we decided to calibrate all HMRs from any collimators to a ME general-purpose (MEGP) collimator condition. This resulted in a CC of 0.88 (ME88 condition). The conversion equation is as follows:

$${\text{HMR}}_{\text{std}} = \left\{ {\frac{{{\text{CC}}_{\text{std}} }}{{{\text{CC}}_{\text{institution}} }}} \right\}\,*({\text{HMR}}_{\text{institution}} - 1) + 1,$$

where HMR_std_ is the standardized HMR, CC_std_ is 0.88, CC_institution_ is the CC for the specific institutional camera–collimator system, and HMR_institution_ is the institutional measured HMR, respectively.

**Fig. 3:**
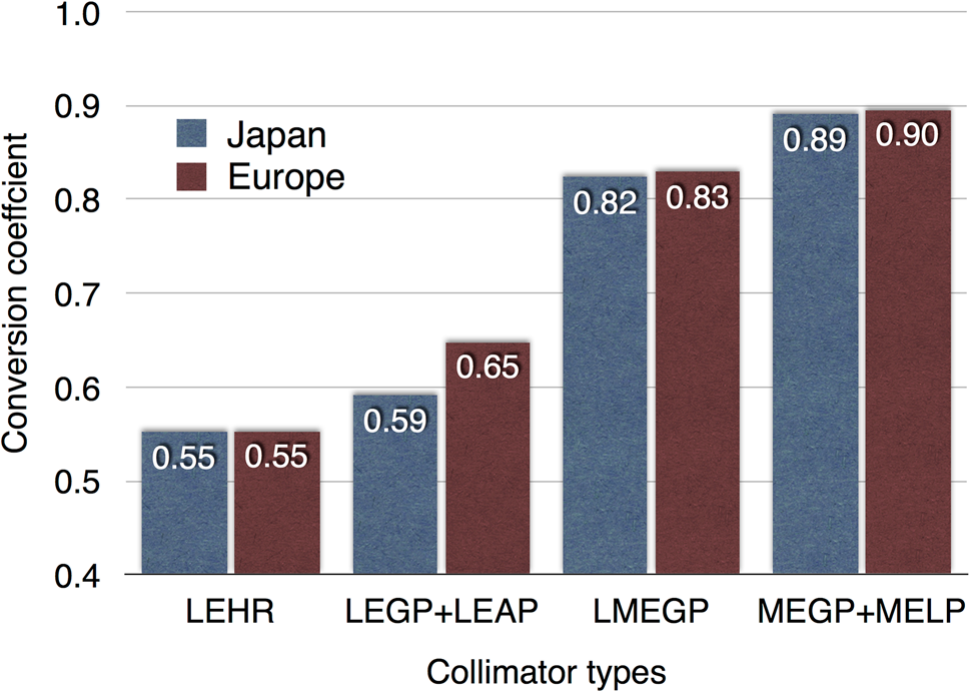
Conversion coefficients obtained in Japanese and European centers [16, 27]

The conversion coefficients measured in Japanese and European studies are summarized in Fig. 3 [16, 27]. There was a good agreement between Japanese and European LE high-resolution (LEHR), low–medium-energy (LME) and MEGP collimators. This finding is most likely a good basis for comparing the data from different camera–collimator systems worldwide. It should be noted that collimator designs are different among companies. Although this variation in CC is smaller for gamma-camera–collimator combinations from the same vendors, differences still exist for the contemporary camera types [27]. This is illustrated by, for example, the extended low-energy general-purpose collimator (GE Healthcare). This collimator showed different CC (0.62 ± 0.03 and 0.75 ± 0.03) depending on camera types used. The differences could be explained by the fact that collimator specifications were modified while maintaining the same collimator name. Applicability of the camera–collimator-specific CC should be further validated in the United States.

Using the CC of the ME88 condition, average and normal range of HMR including both ME and LE collimators became 3.1 (range 2.2–4.0) for early HMR and 3.3 (range 2.2–4.4) for late HMR in JSNM-WG databases [28].

In addition to the phantom-based approach as shown in this article, several methods have been described to correct for differences between collimators. Multiple energy window methods including triple energy window and dual-energy window methods were used to subtract scatter or penetration fraction [15, 29–31]. These corrections did not completely compensate the error in calculation. Empiric correction between two camera systems could be used in a single center [32], but this approach is limited by the need for additional experiments in other camera–collimator combinations. Chen et al. used ^123^I cardiac SPECT imaging with deconvolution of septal penetration, and significantly improved quantitation in ^123^I cardiac SPECT imaging [33]. They also applied three-dimensional SPECT approach for calculating HMR [34]. However, from a prognostic point of view, HMR obtained from SPECT was equivalent to the planar HMR for differentiating between subjects with normal and abnormal ^123^I-*m*IBG uptake. Compared to these methods, a phantom-based correction is practical and applicable for any camera–collimator combination. The HMR provided by a specific collimator type in one hospital can be adjusted to any other camera–collimator conditions.

## Washout rate

The ^123^I-*m*IBG washout rate (WR) is considered to reflect sympathetic drive in physiological and pathological conditions. It has thus been recognized as a predictor of cardiac events and more specifically as a predictor of sudden cardiac death. WR (%) is defined as:

$$\text{WR = }\left\{ {\frac{{\text{(early H - early M) - (late H - late M)*decay correction factor}}}{{\text{(early H - early M)}}}} \right\}{*100\% ,}$$

where decay correction factor = 1/0.5 ^ (time between early and late imaging (h)/13) [12] (asterisk denotes multiplication). In this formula, depending on the use of background subtraction and decay correction, four possible calculation methods can be used, but applications of both time decay and background corrections are generally recommended. In addition, the duration between early and late imaging varies from 2.5 to 5 h in clinical practice, and the definition of WR varies considerably between studies. These potential factors that influence the WR should be standardized, and where possible, correction to a reference value is required [11, 12]. Despite the differences in the time between the late and early acquisition, a correction method for WR may be feasible.

Compared to WR, the late HMR is relatively consistent in CHF patients from 2 to 4 h after the administration of ^123^I-*m*IBG [35, 36], and even a single early-only imaging may be used particularly in patients with Lewy body disease [37]. To obtain reproducible results, caution is required when myocardial uptake is very low. After subtraction of background, the denominator of the abovementioned equation becomes small, and fluctuation of WR becomes larger. Therefore, the WR should be carefully checked in CHF patients and in patients with Lewy body disease with low myocardial ^123^I-*m*IBG uptake.

Another definition of WR used the following equation: WR (%) = (early HMR − late HMR)/early HMR) * 100 [38–40] (asterisk denotes multiplication). The WR calculated with this definition is influenced by the standardization to the ME88 condition.

## Myocardial ^123^I-*m*IBG SPECT

**Fig. 4:**
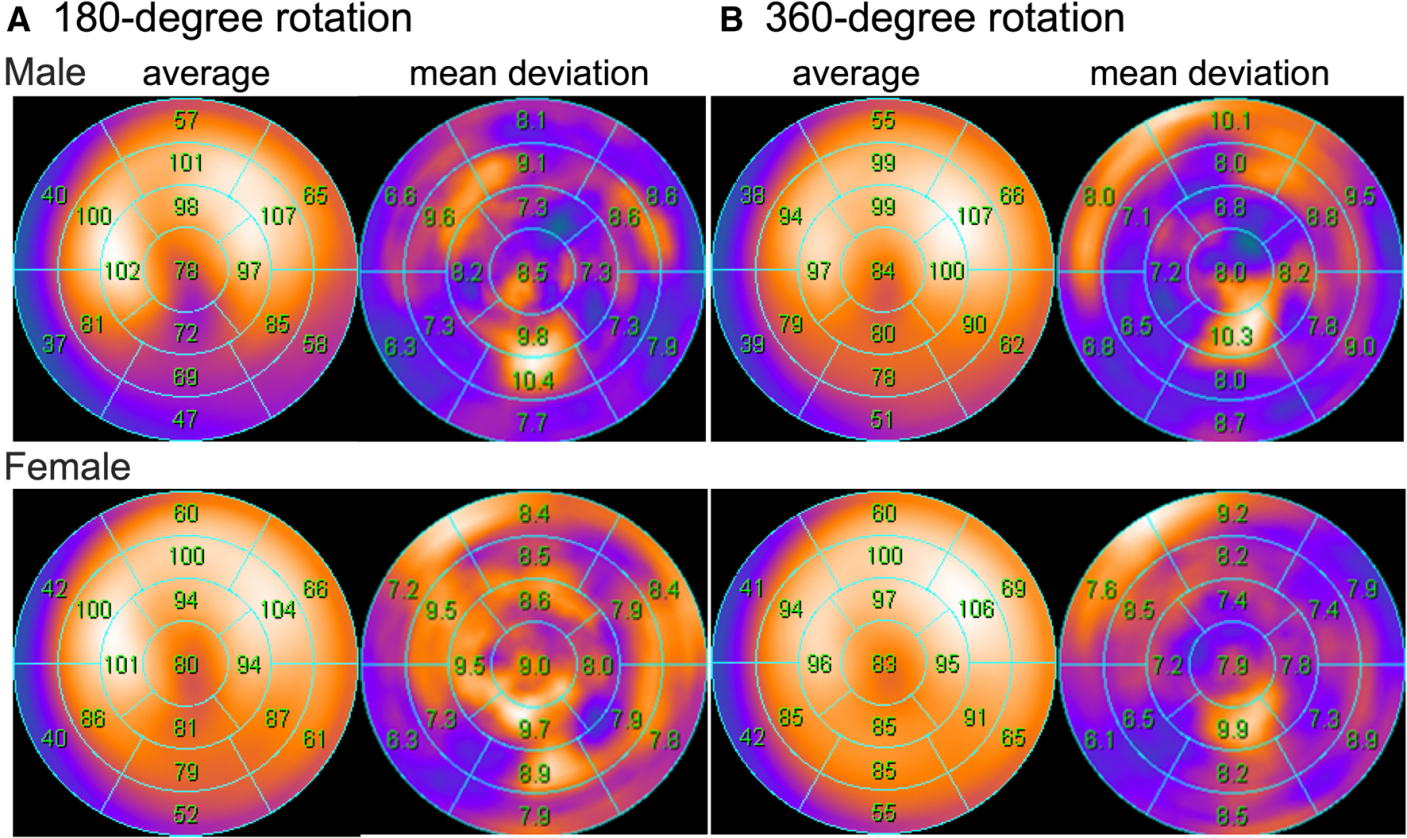
Normal polar plots of the late phase (3–4 h) SPECT ^123^I-*m*IBG images based on the normal ^123^I-*m*IBG databases from the Japanese Society of Nuclear Medicine working group [25, 28]

Defect scoring has been successfully used for myocardial perfusion imaging. The aim of scoring is not only for diagnostic purposes but also to guide therapeutic decision-making. Similar to perfusion studies, the extent and severity of ^123^I-*m*IBG SPECT images can be semi-quantitatively scored. This can be done by a simple visual analysis of the innervation images alone. However, by combining perfusion and innervation images, additional information can be obtained [41]. In most patients with ischemic heart failure, innervation defects are more pronounced than perfusion defects, and the size of mismatch or perfusion–innervation imbalance seems to correlate with the occurrence of serious ventricular arrhythmias [42]. Inhomogeneity of regional sympathetic denervation may be related to serious arrhythmic events and sudden cardiac death. Interestingly, regional myocardial denervation instead of global ^123^I-*m*IBG myocardial uptake was an independent predictor of a positive outcome of electrophysiological studies [43, 44]. To perform this sort of semi-quantitative analysis, normal ^123^I-*m*IBG databases are desirable as a basis for quantitative evaluations. These databases will help us to better understand normal variations in myocardial ^123^I-*m*IBG distribution. For example a decreased uptake in the inferior wall is sometimes observed, particularly in elderly patients. Figure 4 shows polar plots based on the normal databases from the Japanese Society of Nuclear Medicine working group [43]. Mid to apical inferior walls show relatively low counts, particularly in male subjects with 180-degree rotation acquisition. It is also noteworthy that the apical inferior segments on the deviation maps show larger deviation compared with other segments. Based on these normal databases, slightly decreased activity in the inferior regions is not judged as abnormal. These normal databases can help us to define physiological variations during interpretation.

## Mortality risk models

**Fig. 5:**
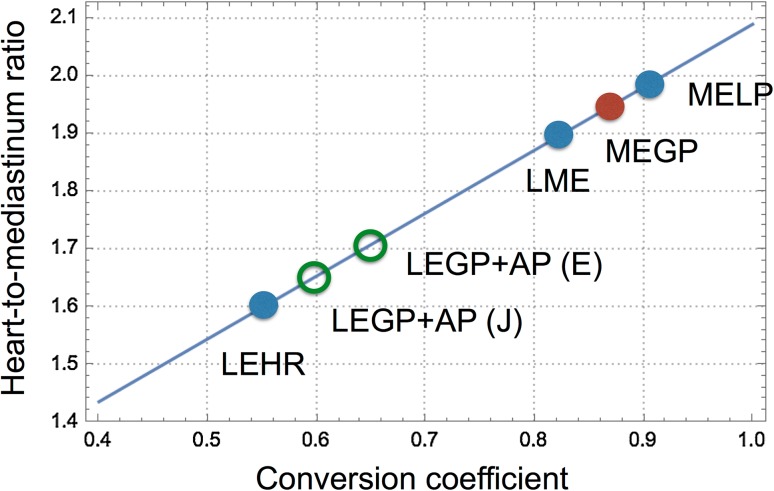
Relationship between heart-to-mediastinum ratio (HMR) and conversion coefficient (CC) using the HMR threshold of 1.6. For example, HMR = 1.6 with a LEHR collimator (CC = 0.55) can be interpreted as 1.9 with the LME collimator (CC = 0.83). Low-energy general-purpose (LEGP) + all-purpose (AP) groups show variable results between Japan (J) and Europe (E). See also average CC in Fig. 3

Standardization of the ^123^I-*m*IBG HMR is especially important in estimating prognosis. After the publication of the ADMIRE-HF study, a HMR cut-off value of 1.6 has often been used for clinical studies in CHF. In this multicenter study, all hospitals used LEHR collimators from several vendors. This cut-off value of 1.6 should be interpreted differently depending on institutional camera–collimator types. As the HMRs for various collimator types are linearly converted versus CC, corresponding HMR values of HMR = 1.6 for the LEHR collimator versus CC are shown in Fig. 5.

**Fig. 6:**
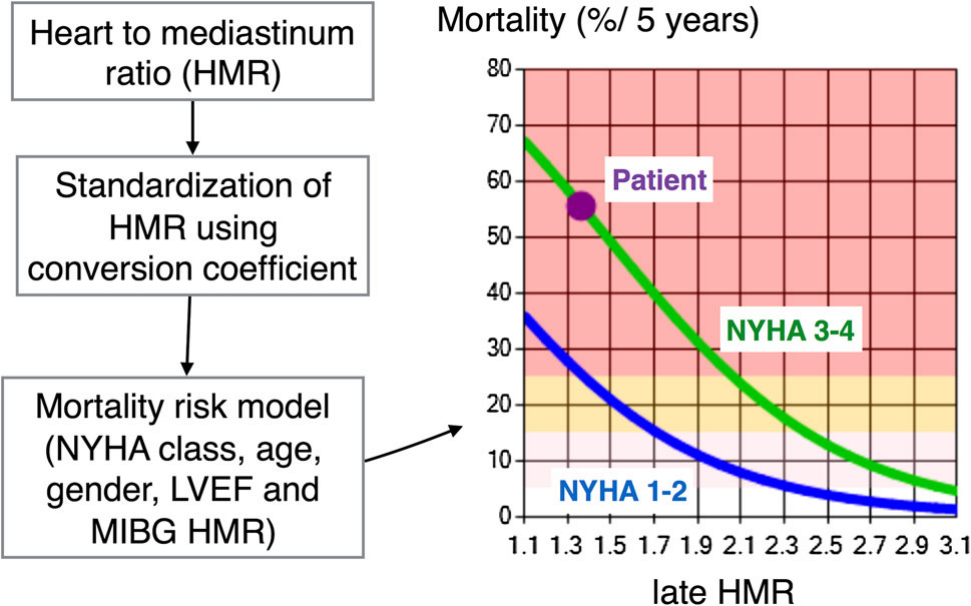
Application of the heart-to-mediastinum ratio (HMR) in a risk model. A sample plot of 5-year cardiac mortality risk is shown in a 54-year-old man with chronic heart failure, which is expressed as a function of HMR. This calculation is based on conditions of NYHA class III, left ventricular ejection fraction of 20%, and HMR of 1.4, and estimated mortality is 56%/5 years (*purple dot*) [45, 46]

More importantly, the risk of cardiac death is multi-factorial, including age, sex, NYHA functional class, left ventricular ejection fraction (LVEF), B-type natriuretic peptide (BNP), and the late ^123^I-*m*IBG HMR [25]. Among these various parameters, we have selected 5 prognostic variables, including the late ^123^I-*m*IBG HMR, and successfully made risk models [45, 46] (Fig. 6). To apply such risk models, HMRs from different acquisition conditions should be standardized so that reliable calculations can be performed.

## Application of standardized HMR in the literature

A number of studies have been performed in cardiology and neurology using the ^123^I-*m*IBG HMR. Although meta-analyses and comparative studies have been performed, standardization is useful to integrate results from various studies. As mentioned previously, averaged HMRs in normal subjects are 2.5 for LEHR collimators and 3.0 for MEGP collimators. Looking at HMR normal values in various Japanese studies from 1994 to 2007, the mean early and late HMRs in the control groups ranged from 1.88 to 2.87 and from 1.84 to 2.49, respectively [45, 46]. In patients with CHF, the best thresholds for differentiating good and bad prognosis and Lewy body and non-Lewy body diseases ranged from 1.5 to 2.1. If the original databases can be accessed with information from the camera–collimator system used, individual HMR data can be corrected. This most likely will reduce the observed variation between the different studies. Even when we compare individual institutional data to the published data, such cross-calibration can be done, and approximate values fitted for individual camera–collimator conditions can be calculated.

## Standardization: perspectives

Ideally, all institutions using myocardial ^123^I-*m*IBG scintigraphy should have their acquisition machines cross-calibrated. This would result in universally applicable cut-off values. In addition, this would result in more uniform risk models. This cross-calibration process will take time. Therefore, it is essential that the currently available data on myocardial ^123^I-*m*IBG scintigraphy of published studies should undergo some form of correction. Ultimately this will make these data easier to implement in clinical practice and will allow for more reliable risk models.

## Conclusion

In CHF, cardiac ^123^I-*m*IBG scintigraphy alone and more likely in combination with other determinants may be able to better select CHF patients with increased risk. Standardization of myocardial ^123^I-*m*IBG outcome parameters is, therefore, essential. This standardization may facilitate a universal implementation of myocardial ^123^I-*m*IBG scintigraphy. The use of a cross-calibration phantom is an effective method for comparing and compiling multicenter databases. In addition, these cross-calibrated HMRs allow for adequate risk models. The role of myocardial ^123^I-*m*IBG scintigraphy in cardiac and neurological applications should be re-evaluated based on such standardized approaches.
